# Synthesis of Multifunctional Polymersomes Prepared by Polymerization-Induced Self-Assembly

**DOI:** 10.3390/polym15143070

**Published:** 2023-07-17

**Authors:** Hien Phan, Robert Cavanagh, Philippa Jacob, Damien Destouches, Francis Vacherot, Benedetta Brugnoli, Steve Howdle, Vincenzo Taresco, Benoit Couturaud

**Affiliations:** 1 Institut de Chimie et des Matériaux Paris-Est (ICMPE), CNRS, University Paris Est Créteil, UMR 7182, 2 Rue Henri Dunant, 94320 Thiais, France; thi-dieu-hien.phan@cnrs.fr; 2 School of Chemistry, University of Nottingham, University Park, Nottingham NG7 2RD, UK; philippa.jacob@nottingham.ac.uk (P.J.); benedetta.brugnoli@uniroma1.it (B.B.); steve.howdle@nottingham.ac.uk (S.H.); vincenzo.taresco@nottingham.ac.uk (V.T.); 3 School of Medicine, University of Nottingham, University Park, Nottingham NG7 2RD, UK; robert.cavanagh@nottingham.ac.uk; 4 TRePCa, University Paris Est Créteil, 94010 Créteil, France; damien.destouches@u-pec.fr (D.D.); francis.vacherot@u-pec.fr (F.V.); 5 Department of Chemistry, Sapienza University of Rome, Piazzale Aldo Moro 5, 00185 Rome, Italy

**Keywords:** polymer-based nanomedicine, polymerization-induced self-assembly (PISA), reactive oxygen species (ROS), hydrogen peroxide (H_2_O_2_), responsive nanoparticles, targeted drug delivery

## Abstract

Polymersomes are an exciting modality for drug delivery due to their structural similarity to biological cells and their ability to encapsulate both hydrophilic and hydrophobic drugs. In this regard, the current work aimed to develop multifunctional polymersomes, integrating dye (with hydrophobic Nile red and hydrophilic sulfo-cyanine5-NHS ester as model drugs) encapsulation, stimulus responsiveness, and surface-ligand modifications. Polymersomes constituting poly(*N*-2-hydroxypropylmethacrylamide)-*b*-poly(*N*-(2-(methylthio)ethyl)acrylamide) (PHPMAm-*b*-PMTEAM) are prepared by aqueous dispersion RAFT-mediated polymerization-induced self-assembly (PISA). The hydrophilic block lengths have an effect on the obtained morphologies, with short chain P(HPMAm)_16_ affording spheres and long chain P(HPMAm)_43_ yielding vesicles. This further induces different responses to H_2_O_2_, with spheres fragmenting and vesicles aggregating. Folic acid (FA) is successfully conjugated to the P(HPMAm)_43_, which self-assembles into FA-functionalized P(HPMAm)_43_-*b*-P(MTEAM)_300_ polymersomes. The FA-functionalized P(HPMAm)_43_-*b*-P(MTEAM)_300_ polymersomes entrap both hydrophobic Nile red (NR) and hydrophilic Cy5 dye. The NR-loaded FA-linked polymersomes exhibit a controlled release of the encapsulated NR dye when exposed to 10 mM H_2_O_2_. All the polymersomes formed are stable in human plasma and well-tolerated in MCF-7 breast cancer cells. These preliminary results demonstrate that, with simple and scalable chemistry, PISA offers access to different shapes and opens up the possibility of the one-pot synthesis of multicompartmental and responsive polymersomes.

## 1. Introduction

Cells are the fundamental building blocks of life, compartmentalizing essential organelles and genetic materials within a bilayer cell membrane [1]. The bilayer acts as a barrier, allowing for a selective passage of molecules via its intermediate protein channels into and out of the cells [1]. Such a critical biological nature inspired a plethora of research to generate synthetic analogues that could mimic living cellular structures and functions, while being simple to produce and tailor for further beneficial applications, particularly in drug delivery [2,3,4,5]. One of representative examples is polymeric vesicles, or polymersomes that are composed of a hydrophilic core within a hydrophobic layer [6,7,8,9]. Therefore, polymersomes enable the convenient entrapment of hydrophobic molecules in the bilayer membrane and hydrophilic payloads within the aqueous lumen [10,11,12,13,14]. Furthermore, a large selection of monomers for synthesizing the building blocks, as well as a fine control over their molecular weights, can impart tunable physicochemical properties, such as stability and permeability, along with chemical diversity, to functionalize with ligands for targeted drug delivery [15,16].

Polymersomes are typically made from AB diblock, ABA triblock, BAB triblock, or ABC triblock copolymers. However, the AB diblock is the most prevalent and well-established composition to form polymersomes, owing to its simpler architecture [17]. All these block copolymers can self-assemble into polymersomes via several techniques, such as hydration, electroformation, emulsion phase transfer, and polymerization-induced self-assembly (PISA) [12,17,18]. Among these, PISA directly combines the synthesis of the block copolymer and its self-assembly in one pot, which removes multiple intermediate steps, while enabling control over particle sizes and shapes [19,20,21,22]. PISA can also produce suspensions with very high final polymersomes concentrations of up to 50 wt%, rendering potential scale-up processes compatible with current standards in the pharmaceutical industry [23,24,25].

In addition to drug loading, polymersomes can be engineered for controlled drug release. Specifically, polymersomes are encoded with functionalities that can respond to abnormal parameters in diseased tissues, such as the pH, redox potential, or the presence of specific enzymes [26,27,28,29] Among these parameters, systems that are sensitive to reactive oxygen species (ROS) have garnered increasing interests due to their implications in various diseases. They are endogenously produced and include hydrogen peroxide (H_2_O_2_), hydroxyl radical (OH^●^), and superoxide (O_2_^−^). ROS at moderate concentrations are essential for normal cell functions, while increased amounts are associated with various pathological disorders, such as cancers, infections, diabetes, and neurodegenerative diseases [30,31,32]. For example, hypoxia, as one of cancer’s distinctive characteristics, leads to a substantial modification of ROS levels within tumor tissue, resulting in cancer cells exhibiting much higher ROS levels (up to 100 × 10^−6^ M) compared to normal tissue (approximately 20 × 10^−9^ M) [33]. Oxidation-responsive functionalities have been incorporated into polymersomes, which at high concentration of ROS, exhibit the structural modification of the nano-assemblies to release the internal payloads [34,35,36].

Recently, we synthesized a novel H_2_O_2_-responsive monomer, *N*-(2-(methylthio)ethyl)acrylamide (MTEAM), which was polymerized with a poly(ethylene glycol) methacrylate-based macro-chain transfer agent (macro-CTA) by aqueous dispersion, RAFT-PISA. The P(PEGMA)-*b*-PMTEAM resulted in spherical micelles and vesicles. However, only spherical particles were degraded at 1–10 mM in H_2_O_2,_, arising from a solubility transition of the MTEAM core from hydrophobic thioethers to hydrophilic sulfoxides [37]. On the other hand, the vesicle form remained relatively stable under the same oxidative conditions. Considering the benefits of polymersomes in drug delivery, the present study attempts to produce polymersomes degraded by an increase of H_2_O_2_. We hypothesized that the type of macro-CTA or hydrophilic block used may affect the resulting NPs’ responses to H_2_O_2_. Therefore, we replaced PEGMA with another widely accessible macro-CTA, poly(*N*-2-hydroxypropyl methacrylamide), referred to as P(HPMAm). The HPMAm moiety was selected, since it is smaller and less bulky than PEG. It also exhibits the potential for further conjugation, non-immunogenicity, and good safety in Phase I clinical trials [38,39,40,41]. This work will thus focus on the improvement in terms of shape/architecture, self-assembling, and responsiveness obtained by moving from PEGMA to HPMAm.

Additionally, we aimed to prepare targeted polymersomes by PISA. Folate receptors are abundantly present on many cancer cell membranes, such as in breast, ovarian, lung, kidney, or brain tumors [42,43,44]. Folic acid (FA), displays a great affinity toward folate receptors. Hence, the conjugation of FA onto NPs promotes receptor-mediated endocytosis and enhances specific cellular uptake [45,46,47].

As a whole, this study aims to investigate the effects of the hydrophilic block on the resulting particle morphologies and to assess the drug release of the obtained polymersomes in increasing H_2_O_2_. Finally, the macro-CTA is functionalized with FA, which is chain-extended with the MTEAM monomer, resulting in polymersomes with a proof-of-concept folate-targeted effect and ROS-responsive drug release.

## 2. Results and Discussion

### 2.1. Synthesis of the P(HPMAm) Macro-CTA

**Figure 1:**
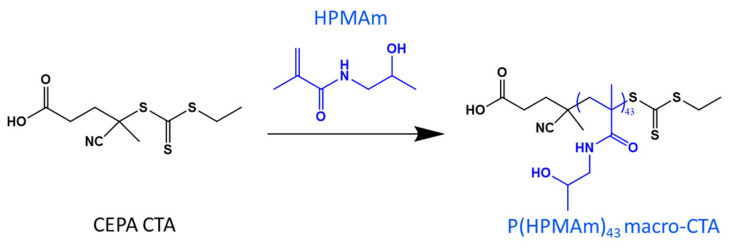
Diagram of RAFT polymerization of HPMAm in acetate buffer (pH 5.5)/dioxane (4/1), yielding P(HPMAm)_43_ homopolymer at 180 min.

The P(HPMAm) macro-CTA used in this study (see Figure 1) was prepared as previously reported, by the chain extension of 4-cyano-4-(((ethylthio)carbonothioyl)thio)pentanoic acid (CEPA) CTA with the HPMAm monomer by RAFT polymerization in a solvent mixture comprising acetate buffer at pH 5.5 and dioxane, at a volume ratio of 4 to 1 and with a monomer initial concentration of 1 mol L^−1^ [41]. The addition of an acetic buffer aimed to prevent the essential dithioester chain-ends from hydrolyzing during the polymerization. The corresponding reaction scheme is shown in Figure 1, and characteristic features of the reaction kinetics are displayed in Figure S1. As such, the semi logarithmic plots exhibit a decent linear correlation trend, indicating that the polymer evolved periodically over the entire reaction time, with little or no termination, as expected from a RAFT polymerization (Figure S1A). In addition, SEC chromatograms show a regular increase in molecular weights during the polymerization time. The chromatograms are generally unimodal and symmetrical, with a relatively small dispersity of 1.2, maximum (Figure S1B), conferring a controllable homopolymerization and a defined polymer structure with desirable chain branching, as similarly observed in the previous work [41]. To suit the subsequent purposes of this work, two macro-CTAs with different chain lengths were synthesized, a shorter chain P(HPMA)_16_ and a longer chain P(HPMA)_43_; both were obtained after 180 min of reaction. The indices (16 and 43) refer to the DPs as measured by ^1^H NMR, using characteristic signals from the end-groups and repeat monomer units.

### 2.2. Synthesis of the P(HPMAm)-b-P(MTEAM) Block Copolymer Carriers

**Figure 2:**
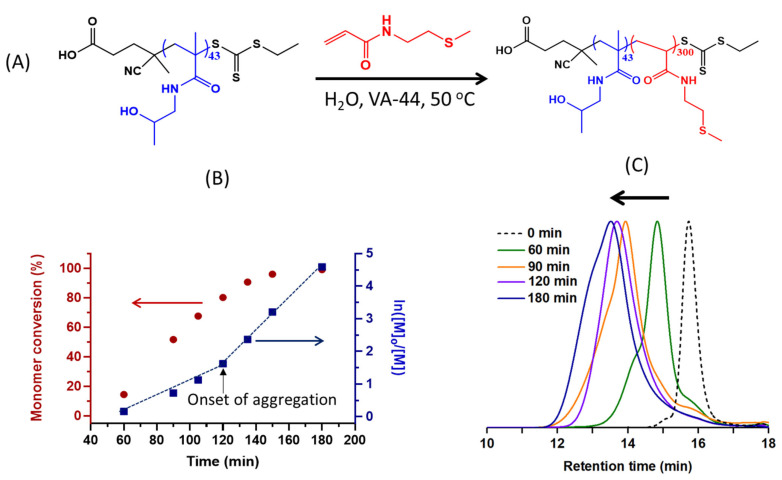
Preparation of block copolymer NPs using aqueous dispersion PISA. (**A**) Reaction scheme for the polymerization of MTEAM monomer using P(HPMAm)_43_ macro-CTA; (**B**) monomer conversion over time and corresponding semilogarithmic plots; (**C**) SEC chromatograms.

The P(HPMAm)_43_ macro-CTA prepared above was chain extended with a previously synthesized ROS-sensitive MTEAM monomer (Figure 2A), using dispersion RAFT-mediated PISA in deionized water, targeting a DP of 300 and 10 wt% solid contents. A summary of the data associated with the PISA reaction, including monomer conversion (%), experimental DPs, molecular weights from ^1^H NMR and SEC, along with particle sizes, is included in Table 1, with some key areas graphically displayed in Figure 2B,C. The semilogarithmic plots (first-order kinetics, Figure 2B) show that the reaction evolves in two separate stages, with a transition from the first to the second stage observed at about 120 min, indicating that the critical onset of aggregation starts at that point. Prior to this time, the block copolymer generates particles, as indicated by DLS, with particle sizes increasing from 90 nm to 109 nm from 60 min to 105 min, but with very high PDI values ranging from 0.3 to 0.5 (Table 1). These results demonstrate that some NPs are formed, but are not yet stable and well-defined. In the 105–120 min range, NPs are sized at 113 nm, with a lower PDI of 0.26. In the later stage, after 120 min, particle sizes increase slightly, to 117 nm at 180 min (Table 1), and particles become more stable, with a reasonable PDI of 0.25. The observed size evolution corroborates with the increase in molecular weights, as determined by SEC (Figure 2C). The chromatograms are relatively unimodal, with progressive deviations resulting from the heterogeneous polymer growth. They evolve rapidly from 0 min to 90 min, during which particles are forming, then increase more slowly, parallel to the stabilizations in particle sizes discussed above.

### 2.3. Fabrication of Vesicles and Oxidative Behavior in H_2_O_2_ Solutions

**Figure 3:**
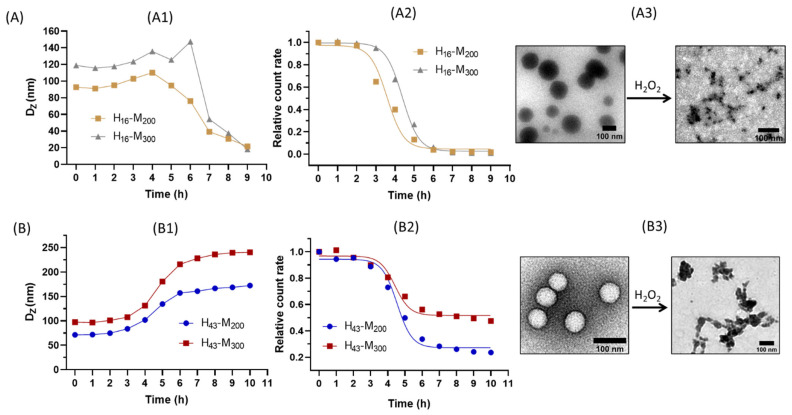
Characteristics of H_16_-M_n_ (**A**) and H_43_-M_n_ (**B**). Sizes (**A1**,**B1**) and relative count rates (**A2**,**B2**) according to DLS; TEM (**A3**,**B3**) of H_16_-M_300_ and H_43_-M_300_, respectively, before and after 10 h of incubation with 10 mM H_2_O_2_.

The purpose of this experiment is to investigate the influence of the hydrophilic chain length and hydrophobic block on the particle sizes, shapes, and ultimately, on their responses, such as disintegration or aggregation in H_2_O_2_ solutions. Two macro-CTAs with short chain P(HPMAm)_16_ and long chain P(HPMAm)_43_ were reacted with MTEAM monomer, targeting DPs of 200 and 300 for the second block. Four block copolymer NPs were obtained accordingly, i.e., P(HPMAm)_16_-*b*-P(MTEAM)_200_ (shorted as H_16_-M_200_) and P(HPMAm)_16_-*b*-P(MTEAM)_300_ (H_16_-M_300_) (Group 1, Figure 3A) and P(HPMAm)_43_-*b*-P(MTEAM)_200_ (H_43_-M_200_) and P(HPMAm)_43_-*b*-P(MTEAM)_300_ (H_43_-M_300_) (Group 2, Figure 3B).

Before incubating with H_2_O_2_ and using the same hydrophilic chain length, increasing DP from 200 to 300 induces an increase in the thickness of the hydrophobic layer, thus resulting in larger particle sizes. This is in agreement with trends previously described in the literature [48,49,50,51,52]. When maintaining the hydrophobic block at a DP of 300, shorter chain copolymers P(HPMAm)_16_ generate spherical particles (Figure 3(A3)), while longer chain copolymers P(HPMAm)_43_ produce vesicles (Figure 3(B3)). Although the PISA systems reported in the literature indicate that a wide range of morphological variations can be obtained, there have only been a few examples of systems ceasing at a spherical particle step and not extending to the formation of vesicles, and the underlying reasons is currently unknown [53,54,55]. In another context, Lesage de la Haye et al. described that poly(*N*-acryloylmorpholine)-*b*-polystyrene (PNAM_26_-*b*-PS_90_) only induced spheres, but when including three poly(ethylene glycol acrylate) (PEGA) units into the (PNAM_26_-*b*-PS_90_) copolymer, vesicles were formed [55]. The authors suggested that the addition of PEGA makes the hydrophilic backbone more rigid, which facilitated the transition to vesicles. Based on this report, it might be hypothesized that the longer macro-CTA P(HPMAm)_43_ generates a higher number of hydrogen bonds with water, increasing its rigidity and lowering the degree of interfacial curvature between the two blocks, thus leading to higher-ordered vesicle forms.

In the 10 mM H_2_O_2_ environment, the above NPs display different trends. For short H_16_-M_300_, a slight increase in size is observed, followed by a burst into smaller scattered pieces, as presented in the TEM image of Figure 3(A3). The particle size of H_16_-M_300_ increases from 120 to 150 nm after 6 h of treatment, with particles bursting into smaller aggregates (60 nm) after 7 h and continuing to decrease in size down to 20 nm after 9 h. H_16_-M_200_ shows the same tendency, although particle sizes increase faster to a maximum at 4 h and gradually disintegrate to 20 nm after 9 h. The difference in bursting time might result from the different initial DPs and particle sizes and from the fact that larger particles have a thicker hydrophobic layer (DP of 300), resulting in the oxidation reaction requiring more time to erode the particles, which in turns extends the disintegration process, as compared to the requirements of the thinner hydrophobic layer with a smaller DP of 200. Meanwhile, NPs composed of a longer hydrophilic chain, H_43_-M_n_, displayed a completely different evolution process, with particles becoming larger and forming aggregates, the sizes significantly increasing from 100 to 230 nm for H_43_-M_300_ and from 70 to 170 nm for H_43_-M_200_. This change in degradation might be due to the altered permeability of the various polymersomes formed [56,57].

In summary, two separate lengths of the macro-CTAs induced completely different outcomes when oxidized in H_2_O_2_, with the particles in both groups first swelling in the first 6 h, and then evolving in two different directions afterward. The swelling state indicated a permeation by H_2_O_2_ and suggested the effective oxidation of hydrophobic thioethers into hydrophilic sulfoxides, inducing a loss in the compacity of the cores, with the particles appearing loose, swollen, and deformed. The distinct evolutions in the later stage might be due to the thickness of the hydrophilic layers and to the different morphologies. We observed a similar tendency in a previous work [37]. Shorter chain P(HPMAm)_16_ generated thinner external hydrophilic layers, fragilizing the entire particle structure by allowing the oxidation to break down the spherical particles faster. In contrast, longer chain P(HPMAm)_43_ formed thicker and denser external and internal hydrophilic layers, thus limiting the diffusion of H_2_O_2_ and slowing down the degradation process.

A successful oxidative degradation was also confirmed by a substantial decrease in relative count rates in DLS, which indicates that the particles became very diluted, while slowly disappearing (Figure 3(A2,B2)). Figure S2 also shows the visual transformation— which can be observed with a naked eye—of the vesicle suspensions, transitioning gradually from opaque white to transparent over the oxidation process. The zeta potentials of NPs before the incubation with H_2_O_2_ solutions were around—20 mV; they gradually increased to 0–5 mV when incubated with H_2_O_2_ (Figure S3), further confirming that those particles had been modified and had lost their original surface properties.

**Figure 4:**
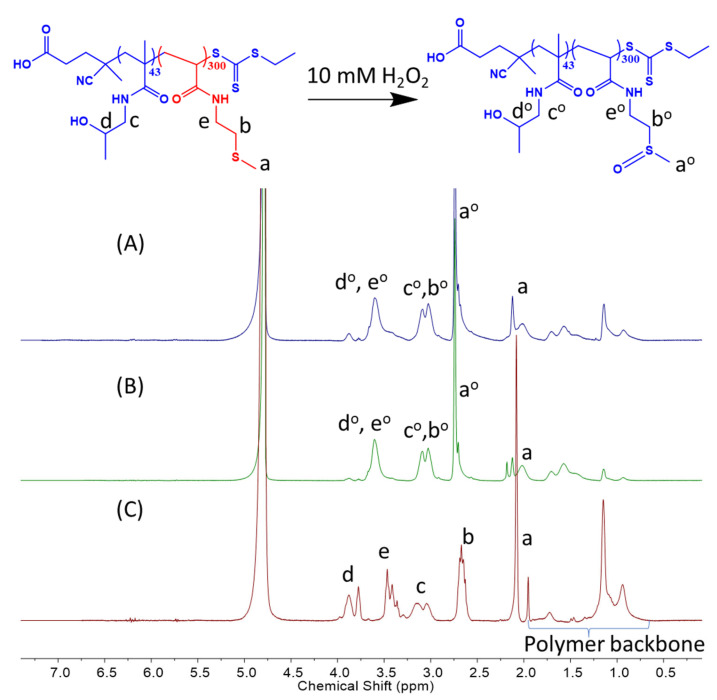
NMR spectra of oxidized H_43_-M_300_ (**A**) and H_16_-M_300_ (**B**), and non-oxidized H_43_-M_300_ (**C**).

The oxidation of H-M polymersomes is confirmed by NMR (Figure 4). A total of 1 mg mL^−1^ of H_16_-M_300_ and H_43_-M_300_ NPs were incubated with 10 mM H_2_O_2_ for 10 h. The oxidized peaks tended to shift toward the left, with a reference to the control spectrum of H_43_-M_300_ NPs in water. In Figure 4A,B, the methyl proton peak adjacent to the thioether group at 2.1 ppm (a) decreased considerably in intensity and mostly shifted downfield to 2.7 ppm (a^o^). The proton peaks of the -CH_2_ group adjacent to the thioether function (b) disappeared and moved to the left, overlapping with the peaks of the -CH_2_ group of HPMAm (c^o^). Similarly, the proton peaks of -CH_2_ in -CH_2_-NH- of MTEAM (e) were shifted downfield and combined with the proton peaks of -CH in HPMAm (d^o^). These observations align with our recent work and other previous reports regarding peak transitions of the thioether-bearing hydrophobic block to sulfoxide-containing segments [37,57,58,59,60,61].

Along with the reduced count rates and modified shapes after H_2_O_2_ incubation, it is certain that both H_16_-M_300_ and H_43_-M_300_ responded to the oxidative condition and transform the hydrophobic thioethers into hydrophilic sulfoxides, leading to first a disruption of amphiphilic balance in the polymersomes and subsequently, to particle disassembly.

Owing to the versatile structure of vesicles, which can facilitate the loading of hydrophilic and hydrophobic drugs, vesicles (H_43_-M_300_) were also selected for encapsulation and ligand functionalization in subsequent studies.

### 2.4. FA-Functionalized P(HPMAm)_43_ Macro-CTA and Diblock Copolymer Synthesis via the PISA Process with the MTEAM Monomer

**Figure 5:**
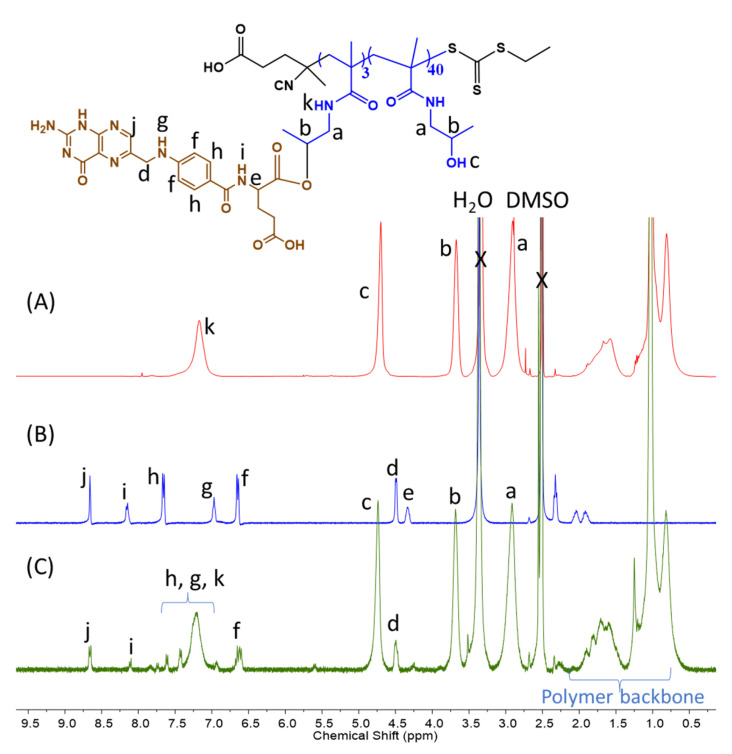
^1^H NMR spectra of: (**A**) initial P(HPMAm)_43_, (**B**) free FA, and (**C**) FA_3_-P(HPMAm)_43_ macro-CTA in DMSO-d_6_.

Some of the hydroxyl groups of the macro-CTA P(HPMAm)_43_ were first functionalized with one of the two carboxylic acid moieties available on FA using the Steglich esterification reaction. FA was attached at a 0.06 mole fraction according to ^1^H NMR data (Figure 5), yielding 3 FA molecules attached per oligomeric macro-CTA. The resulting modified CTA and the subsequent block will be referred to as FA_3_-P(HPMAm)_43_.

**Figure 6:**
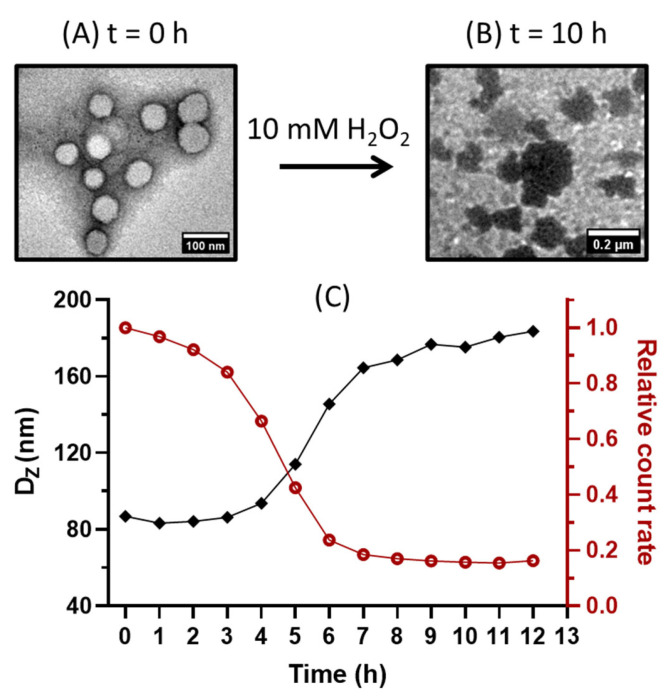
FA_3_-P(HPMAm)_43_-*b*-P(MTEAM)_300_ (F_3_H_43_-M_300_) polymersomes when treated with 10 mM H_2_O_2_. (**A**) at t = 0 h, defined vesicle form; (**B**) at t = 10 h, aggregates; (**C**) DLS-measured sizes and relative count rates.

The FA_3_-P(HPMAm)_43_ macro-CTA was then chain-extended with the MTEAM monomer, targeting a DP of 300. The PISA reaction was undertaken in a mixture of water:ethanol at 7:3 (*v*:*v*) to enhance the solubility of the FA-containing macro-CTA. The polymerization was quenched after 5 h, resulting in FA_3_-P(HPMAm)_43_-*b*-P(MTEAM)_300_ (abbreviated as F_3_H_43_-M_300_) vesicles with a diameter of 90 nm, PDI of 0.07, and ZP of −28 mV (DLS). The corresponding TEM image is provided in Figure 6A. The F_3_H_43_-M_300_ vesicle was submitted to the same degradation protocol with H_2_O_2_ solution, as previously described for plain vesicles. The same sensitivity to H_2_O_2_ was observed, in which particle disassembly, size modifications, and relative count rates over time follow the same tendencies as those observed for the plain vesicles (Figure 6B,C). This result suggested that FA-functionalized polymersomes were sufficiently oxidized to disassemble the nanostructures, leading to reduced count rates and aggregation.

### 2.5. Encapsulation and Release Studies

The hydrophobic dye Nile red (NR) and the hydrophilic dye sulfo-cyanine5-NHS ester (Cy5) were entrapped into folate-targeted polymersomes during PISA. The NR-loaded targeted polymersomes were 90 nm in size, with a PDI 0.21. An NR loading of 0.04 wt% was achieved under the tested conditions, resulting in an encapsulation efficiency (EE) of 72%. Cy5 was loaded at 1.72 µg mL^−1^ with 0.05 wt% of DL and 23% of EE, and the Cy5-loaded vesicles were sized at 140 nm, with a PDI 0.25. This indicated that the FA-functionalized polymersomes enabled the encapsulation of both hydrophilic and hydrophobic compounds.

**Figure 7:**
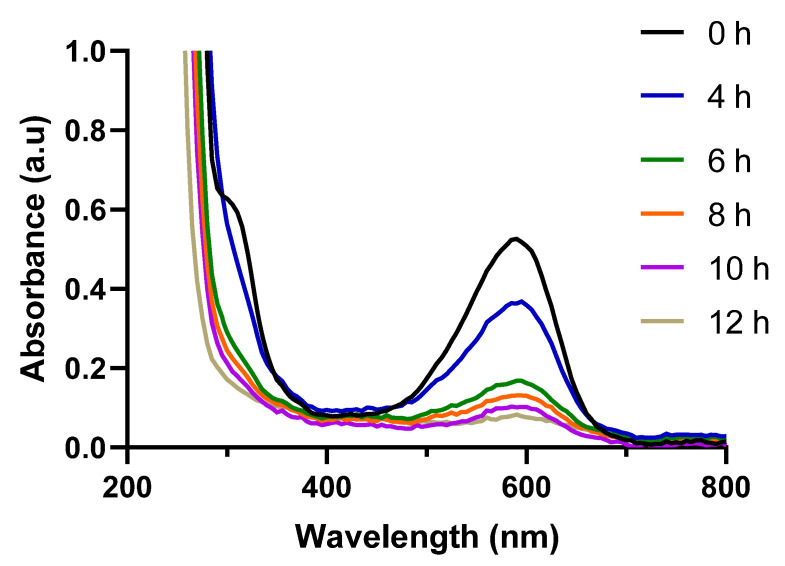
UV-Vis spectra of NR-loaded FA-bound H_43_-M_300_ vesicles when placed in a 10 mM H_2_O_2_ solution.

The release study was undertaken using NR dye, since it is a UV-Vis active fluorescent dye, and it has been largely utilized to investigate drug releases from particles in oxidative environments due to its stable fluorescence and absorbance in these settings [62,63]. Similar to the results of our previously published work [37], a decrease in the absorbance was observed over time when the vesicles were dropped into a 10 mM H_2_O_2_ solution (Figure 7). This progressive disappearance results from the fact that NR is insoluble in water, and mostly precipitates at the bottom of the cuvette when released from the vesicle layer, not subsequently contributing to the total absorbance. As a result, the absorbance adequately describes the amount of NR enclosed in the vesicle layer, whose concentration reduces due to the vesicle degradation process.

### 2.6. Stability in Bio-Relevant Conditions

**Figure 8:**
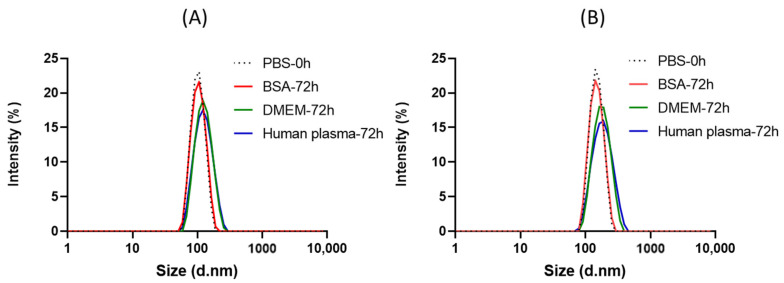
Stability of H_43_-M_300_ (**A**) and F_3_H_43_-M_300_ (**B**) polymersomes in PBS at t = 0 h, and BSA, DMEM, human plasma at t = 72 h.

Ligand-free plain vesicles made up of H_43_-M_300_ and F_3_H_43_-M_300_ were treated with PBS, BSA, DMEM cell culture medium, and human plasma for 72 h and at 37 °C to assess their stability under bio-mimicking conditions (Figure 8). NPs in PBS at 0 h were used as a control. The size distributions of both H_43_-M_300_ and F_3_H_43_-M_300_ polymersomes are nearly unchanged when treated in three biological media over 72 h, with sizes and PDIs of 108 nm, 0.12, and 130 nm, 0.17, respectively. This result demonstrates the significant stability of the obtained polymersomes in both in vitro and human plasma environments, showing their suitability as a very promising drug delivery platform for a safe and effective transition to future clinical applications.

### 2.7. Cytotoxicity

**Figure 9:**
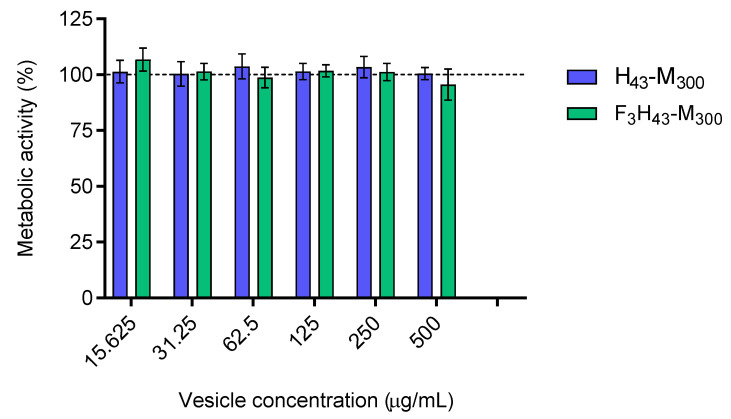
Cytotoxicity of H_43_-M_300_ and F_3_H_43_-M_300_ vesicles on MCF-7 breast cancer cells. Particles were incubated with cells for 24 h and assayed with the PrestoBlue metabolic viability experiment. Experiments were performed in triplicate (3 wells/sample) at three different time points. Data are presented as mean ± S.D.

The toxicity of H_43_-M_300_ and F_3_H_43_-M_300_ polymersomes was assessed on MCF-7 luminal breast cancer cells (Figure 9). Vesicles were applied for 24 h at concentrations up to 500 µg mL^−1^, and then cellular metabolic activity was measured as an indicator of cell viability. The data in Figure 9 demonstrates that all vesicles formed are non-toxic ≤ 500 µg mL^−1^.

## 3. Conclusions and Perspectives

Replacing PEGMA with HPMAm yielded polymersomes that were sensitive to high concentrations of H_2_O_2_. Furthermore, tuning the P(HPMAm) block length allowed access to multiple particle shapes. At the same, P(MTEAM) hydrophobic block length impacted the results, with the short chain of P(HPMAm)_16_ promoting spheres, and the long chain of P(HPMAm)_43_ forming vesicles. This can probably be attributed to the enhanced rigidity and lower interfacial curvature of the P(HPMAm)_43_-*b*-P(MTEAM)_300_ block copolymers, resulting in higher-order morphology, such as vesicles. The different hydrophilic lengths first led to various morphologies, which in turn responded differently when treated with a high concentration of H_2_O_2_. The P(HPMAm)_16_-*b*-P(MTEAM)_300_ spheres fragmented, while the P(HPMAm)_43_-*b*-P(MTEAM)_300_ vesicles aggregated. Either way, this structural modification demonstrated that particles were oxidized in 10 mM H_2_O_2_, owing to the solubility transformation of the thioether groups into the sulfoxide groups. FA-conjugated P(HPMAm)_43_-*b*-P(MTEAM)_300_ vesicles could encapsulate both hydrophilic Cy5 dye and hydrophobic NR dye, which both displayed a programmed release of NR dye in the oxidative environment. Both ligand-free and FA-conjugated polymersomes were stable in PBS, DMEM cell culture medium, and human plasma. All polymersomes formed showed no or low toxicity when tested in in vitro assays against on MCF-7 cells. The current work has provided a proof-of-concept aspect for designing new chemistry that can push PISA toward one-step multicompartment, responsive, and targeted polymersomes. Targeting in vitro and in vivo experiments are ongoing.

## 4. Materials and Methods

### 4.1. Materials

All chemicals were used as obtained, without further purification, unless otherwise stated. The 4-Cyano-4-(((ethylthio)carbonothioyl)thio)pentanoic acid (CEPA) chain transfer agent (CTA) was synthesized according to the established protocol [64]. The chemicals were sourced as follows: sodium carbonate (>95%, Aldrich, St. Louis, MO, USA), 1-amino-2-propanol (>95%, Aldrich), methacryloyl chloride (≥97%, Aldrich), 2,2′-Azobis(2-methylpropionamidine) dihydrochloride azo initiator (VA-50, MW = 271.19 g mol^−1^, ≥97%, Aldrich), 2,2′-Azobis(2-(2-imidazolin-2-yl)propane)dihydrochloride (VA-44, MW = 323.27 g mol^−1^, ≥97%, Aldrich), 2-(methylthio)ethylamine (95%, Thermo Scientific, Waltham, MA, USA), 1,4-dioxane (anhydrous ≥ 99.9%, Aldrich), folic acid (≥97%, Aldrich), 4-(dimethylamino)pyridine (DMAP, ≥97%, Aldrich), dicyclohexylcarbodiimide (DCC, 99%, Aldrich), Nile red (NR, ≥97%, Aldrich), acryloyl chloride (≥97%, Aldrich), triethylamine (TEA, 99%, Aldrich), phosphate buffered saline (PBS, Aldrich), bovine serum albumin (BSA, Thermo Fisher Scientific), Dulbecco’s Modified Eagle Medium (DMEM, Thermo Fisher Scientific), fetal calf serum (FBS, Sigma-Aldrich, St. Louis, MO, USA), PrestoBlue cell viability assay (Thermo Fisher Scientific), l-glutamine (Sigma-Aldrich), Sulfo-Cy5 NHS (Lumiprobe, Cockeysville, MD, USA). MCF-7 human breast adenocarcinoma cells were obtained from the American Type Culture Collection (Manassas, VA, USA) and used in a passage window of 30–35. The qualified plasma was obtained from the Etablissement Français du Sang, and kindly provided by the TRePCa team from UPEC.

### 4.2. Methods

#### 4.2.1. Synthesis of N-2-Hydroxypropyl Methacrylamide (HPMAm)

HPMAm was synthesized by following an established protocol, with a few modifications [65,66,67]. Sodium carbonate (23.3 g, 0.22 mol) was dispersed in 100 mL of dry dichloromethane (DCM) in a round-bottom flask. The mixture was cooled to −10 °C in an ice-acetone bath, and 1-amino-2-propanol (15.4 mL, 0.2 mol) was added. Methacryloyl chloride (19.5 mL, 0.2 mol) was diluted in 20 mL of dry DCM and added dropwise to the previous mixture at −10 °C. The reaction was left at room temperature and stirred for 24 h. The white solid was removed by filtration, and the obtained solution was dried over MgSO_4_ and then filtered. The final solution containing DCM was evaporated under reduced pressure until about 50 mL of DCM was left in the round-bottom flask. This solution was placed in a −28 °C freezer overnight to crystallize. The obtained solid was filtered, dissolved in 50 mL of acetone, and recrystallized overnight, yielding white crystals with a 71% (25 g) yield in the following ratio: ^1^H NMR (D_2_O, 400 MHz, ppm): δ 5.57 (s, 1H, C=CH_2_), 5.32 (s, 1H, C=CH_2_), 3.80–3.86 (m, 1H, C(OH)H), 3.12–3.17 (m, 2H, NH-CH_2_), 1.8 (s, 3H, C=C-CH_3_), 1.05–1.03 (d, 3H, CH(OH)-CH_3_).

#### 4.2.2. Synthesis of the *N*-(2-(Methylthio)ethyl)acrylamide (MTEAM) Monomer

The MTEAM monomer was synthesized according to our previously published protocol [37]. A total of 1.0 g (11.1 mmol, 1.0 mL) 2-(methylthio)ethylamine was added to a round-bottom flask containing 50 mL dry THF and then cooled to 0 °C. A total of 1.1 g (11.1 mmol, 1.5 mL) triethylamine was then added to the solution and left to stir for 10 min. A 0.1 g (11.1 mol, 0.9 mL) portion of acryloyl chloride was added dropwise, and the solution was allowed to warm to room temperature. After 12 h, the white precipitate was filtered, and the THF was removed under reduced pressure. The crude mixture was dissolved in 50 mL of DCM and washed with saturated NaHCO_3_ (2 × 50 mL) and brine (50 mL). The organic layer was dried over anhydrous MgSO_4_, filtered, and the solvent was evaporated in a rotovap. The obtained oil was purified by column chromatography on silica gels, initially using 100% cyclohexane, then cyclohexane:dichloromethane = 50:50 (*v*/*v*), and slowly progressing to dichloromethane:methanol = 98:2 (*v*/*v*), resulting in a colorless oil at a 70% yield.

#### 4.2.3. Synthesis of the P(HPMAm) Macro-CTA

P(HPMAm) was synthesized using RAFT polymerization in a mixture of acetate buffer pH 5.5 and dioxane (4/1, *v*/*v*), aiming at achieving a degree of polymerization (DP) of 60 and using an initial monomer concentration of 1 mol L^−1^, as adopted from a previous protocol [41]. A total of 1.46 g HPMA (10.22 mmol, Eq. 60), 0.04 g CEPA RAFT agent (170.45 µmol, Eq. 1), and 4.6 µg VA-50 (17.04 µmol, Eq. 0.1) were dissolved in 10.22 mL of the buffer/dioxane solvent mixture in a vial sealed with a rubber septum. Since a small quantity of VA-50 was to be added into the reaction mixture, a stock solution of VA-50 was first prepared in the amount of 1 mg mL^−1^ in the solvent solution. The reaction vessel was degassed by gentle bubbling with an argon stream for 30 min and placed in a pre-heated water bath at 70 °C. Samples were periodically withdrawn every 30 min for kinetics study and assessed by ^1^H NMR (DMSO-d_6_) and SEC (poly(methyl methacrylate) (PMMA) standards (DMF). Two macro-CTAs—P(HPMAm)_16_ and P(HPMAm)_43_, obtained after 60 min and 180 min reaction time, respectively—were selected for further study and were purified by dialyzing the obtained solutions against deionized water (molecular weight cut-off = 3500 g moL^−1^) for 48 h, then lyophilizing the resulting aqueous solution, yielding a pale-yellow solid.

#### 4.2.4. Synthesis of P(HPMAm)_n_-*b*-P(MTEAM)_300_ Block Copolymer NPs (*n* = 16, 43)

Block copolymer P(HPMAm)_16_-*b*-P(MTEAM)_300_ and P(HPMAm)_43_-*b*-P(MTEAM)_300_ were prepared by aqueous dispersion RAFT PISA polymerization at 10 wt% of solid content in deionized water. For example, 20 mg P(HPMAm)_43_ (3.05 µmol, Eq. 1), 0.13 g MTEAM (914 µmol, Eq. 300), and 197 µg VA-44 (0.61 µmol, Eq. 0.2) were added to a vial containing 1.53 mL deionized water. To facilitate the addition of small quantity of VA-44, a stock solution of 1 mg mL^−1^ VA-44 was prepared, and an equivalent volume was then added to the reaction mixture. The vial was degassed by gentle bubbling with an argon stream for 30 min and placed in a pre-heated oil bath at 50 °C for 180 min. A polymerization kinetics evaluation was carried out by withdrawing aliquots at predetermined times using degassed syringes and assessing the molecular weights by ^1^H NMR and SEC.

#### 4.2.5. Attachment of Folic Acid (FA) to the P(HPMAm)_43_ Macro-CTA

The conjugation of P(HPMAm)_43_ with FA was obtained by using the Steglich reaction between the hydroxyl functions of P(HPMAm)_43_ and the α-carboxylic acid groups of FA, based on methods used in previous literature, with appropriate modifications [47,68,69]. A total of 0.02 g FA (45.48 µmol, Eq. 5 compared to the macro-CTA) and 0.01 g DCC (46.39 µmol, Eq. 5.1) were dissolved in 4 mL DMSO and stirred at ambient temperature under argon for 24 h. Amounts of 0.06 g P(HPMAm)_43_ (M_n_ = 6596 g mol^−1^, 9.09 µmol, Eq. 1) and 5.55 mg DMAP (45.48 µmol, Eq. 5) were dissolved in 2 mL DMSO and added to the mixture of macro-CTA and DCC, with continuous stirring under argon for 24 h at room temperature. The precipitated by-product dicyclohexyl urea (DCU) was filtered off, and the resulting solution was first dialyzed against DMSO for 24 h to remove the unreacted FA, DMAP, and DCU, then against distilled water for 24 h, using a membrane with a molecular weight cut-off of 3500 g mol^−1^. The obtained solution was freeze-dried overnight, yielding a pale-yellow solid with a 60% yield and a 6% FA attachment, as quantified by ^1^H NMR (DMSO-d_6_).

#### 4.2.6. Preparation of FA-Functionalized NPs

The above FA-bound macro-CTA was chain-extended with the MTEAM monomer, under conditions designed to obtain a DP of 300 and a 5 wt% solid contents of NPs in the suspension. A total of 0.01 g P(HPMAm)_43_-FA_3_ (M_n_ = 7799 g mol^−1^, 1.28 µmol, Eq. 1), 55.9 mg MTEAM (385 µmol, Eq. 300), and 166 µg VA-44 (0.5 µmol, Eq. 0.5) were dissolved in a 1.32 mL solvent mixture of H_2_O:EtOH (7:3) in a vial and then degassed for 30 min. The reaction was placed in a preheated oil bath at 65 °C for 5 h. The formed turbid suspension was filtered via a 0.45 µm Millipore membrane to remove any precipitate and assessed by ^1^H NMR (DMSO-d_6_), DLS and TEM.

#### 4.2.7. Encapsulation of Model Compounds into the FA-Functionalized Vesicles

Nile red was loaded under the PISA conditions used for obtaining FA-bound P(HPMAm)_43_-*b*-P(MTEAM)_300_ at a concentration of 0.1 wt% of NR, with respect to the total solid contents. A stock solution of NR was first prepared at 1 mg mL^−1^ in CHCl_3_. A total of 76 µL of this solution was added in a HPLC 1.5-mL vial, and CHCl_3_ was subsequently evaporated in a vacuum oven. To this vial, 66 mg MTEAM (455 µmol) was added and mixed thoroughly. In another vial, 10 mg FA-bound P(HPMAm)_43_ macro-CTA (1.52 µmol) and 196 µg VA-44 (0.6 µmol) were dissolved in 1.52 mL of a solvent mixture of H_2_O/EtOH at 7/3. The MTEAM/NR mixture was added dropwise to this vial. The sample was degassed by gentle bubbling with an argon stream for 30 min and placed in a pre-heated oil bath at 65 °C. The reaction was quenched by opening the vials to air, and the samples were assessed by DLS and NMR. Free NR was removed by dialyzing the sample against deionized water in a membrane of MWCO = 2000 g mol^−1^ for 48 h. The loaded NR was quantified by UV-Vis by comparison with a known calibration curve of NR in DMSO at 550 nm, as calculated in Formula 1.

Similar to loading NR, the hydrophilic sulfo-Cy5 NHS ester was simultaneously loaded into the folate-targeted polymersomes in H_2_O at 0.1%wt of Cy5 compared to the total weight of solid contents. In brief, a stock solution of Cy5 was prepared at 1 mg mL^−1^ in H_2_O. 56 mg MTEAM (385 µmol), 10 mg FA-conjugated macro-CTA (1.3 µmol), 207 µg VA-44 (0.6 µmol), 66 µL Cy5 stock solution, and 1.23 mL of a solvent mixture made of H_2_O/EtOH at 7/3 were added to a vial. This vial was mixed well, degassed by purging under argon for 30 min, and placed in a pre-heated oil bath at 65 °C. The reaction was quenched by opening the vials to air, and the samples were assessed by DLS and NMR. Free Cy5 was removed by dialyzing the polymersomes obtained against deionized water using MWCO = 2000 g mol^−1^ for 48 h. The loaded Cy5 was quantified by UV-Vis through a known calibration curve of Cy5 in H_2_O at 646 nm, as calculated in Formula (1).

Formula (1). Calculation of Drug Loading (DL) and Encapsulation Efficiency (EE)

(1) $$\mathrm{DL}\;(\%)=\frac{Amount\;of\;drug\;loaded}{Amount\;of\;solid\;contents}\times 100\;\mathrm{EE}\;(\%)=\frac{Amount\;of\;drug\;loaded}{Amount\;of\;drug\;initially\;added}\times 100$$

#### 4.2.8. Cytotoxicity

The cells were cultured in DMEM supplemented with 10% (*v*/*v*) FBS and 2 mM L-glutamine, and at 37 °C with 5% CO_2_. The cytotoxicity of the vesicles was assessed using the PrestoBlue cell viability assay. The MCF-7 cells were seeded at 1 × 10^4^ cells per well in 96-well plates and cultured for 24 h prior to assaying. The vesicles were exposed to the cells for 24 h and added to 100 µL phenol red free DMEM containing 10% (*v*/*v*) FBS and 2 mM L-glutamine. Triton X-100 at 1% (*v*/*v*), applied in the phenol red free medium, was used as a cell death (positive) control, and a vehicle control containing no vesicles was used as a negative control. Following exposure, the cells were washed twice with warm PBS and 100 µL 10% (*v*/*v*) PrestoBlue reagent diluted in a phenol red free medium applied for 60 min per well. The resulting fluorescence was measured at 560/600 nm (λex/λem). The relative metabolic activity was calculated by setting the negative control values as 100% and the positive control values as 0% metabolic activity.

## Figures and Tables

**Table 1:** Summary of characteristic features observed for the RAFT PISA process leading to P(HPMAm)_43_-*b*-P(MTEAM)_300_.

Time (min)	Conv. %	Theoretical DP ^a^	Experimental M_n_ ^b^ (×10^−3^)	Experimental M_n_ ^c^ (×10^−3^)	*Đ*	Size (nm)	PDI
60	14	43	12.7	29.2	1.33	90.44	0.55
90	52	154	35.1	60.1	1.88	111.1	0.44
105	67	202	64.6	69.1	1.86	109.2	0.31
120	80	240	99.5	65.3	1.66	113.1	0.26
135	90	271	138.9	85.0	2.03	113.7	0.25
150	96	287	180.7	89.0	2.12	114.3	0.24
180	96	287	180.6	84.1	2.26	117.3	0.25

^a^ Equal to conversion (%) × 300; ^b^ absolute numbers as measured by ^1^ H NMR; ^c^ relative numbers as measured by SEC using a calibration technique based on PMMA standards.

## Data Availability

Not available.

## Supplementary Materials

The following supporting information can be downloaded at: https://www.mdpi.com/article/10.3390/polym15143070/s1, Figure S1: Kinetics of the RAFT polymerization of HPMAm in acetate buffer (pH 5.5)/dioxane (4/1), yielding P(HPMAm)_43_ homopolymer at 180 min. (A) Monomer conversion % (red) and first-order kinetic plots (blue) as a function of time; (B) SEC chromatogram at 60, 90 and 180 min using DMF and PMMA standard; Figure S2: Color change from turbidity gradually to transparency of P(HPMAm)_43_-*b*-P(MTEAM)_300_ polymersomes (2 mg mL^−1^) when incubated in 10 mM H_2_O_2_ over time; Figure S3: Zeta potentials (ZPs) of (A) P(HPMAm)_16_-*b*-P(MTEAM)_x_ (H_16_-M*_x_*) and (B) P(HPMAm)_43_- *b*-P(MTEAM)_x_ (H_43_-M_x_) (x = 200, 300) polymersomes at 1 mg mL^−1^ during the treatment with 10 mM H_2_O_2_.

## References

[1] Alberts B., Lewis J. (2013). The Lipid Bilayer. Molecular Biology of the Cell.

[2] Honigmann A., Pralle A. (2016). Compartmentalization of the Cell Membrane. J. Mol. Biol..

[3] Derry M.J., Fielding L.A., Armes S.P. (2016). Polymerization-induced self-assembly of block copolymer nanoparticles via RAFT non-aqueous dispersion polymerization. Prog. Polym. Sci..

[4] Peyret A., Ibarboure E., Pippa N., Lecommandoux S. (2017). Liposomes in Polymersomes: Multicompartment System with Temperature-Triggered Release. Langmuir.

[5] Seo H., Lee H. (2021). Recent developments in microfluidic synthesis of artificial cell-like polymersomes and liposomes for functional bioreactors. Biomicrofluidics.

[6] Mohammadi M., Ramezani M., Abnous K., Alibolandi M. (2017). Biocompatible polymersomes-based cancer theranostics: Towards multifunctional nanomedicine. Int. J. Pharm..

[7] Albuquerque L.J., Sincari V., Jäger A., Kucka J., Humajova J., Pankrac J., Paral P., Heizer T., Janouškova O., Davidovich I. (2021). pH-responsive polymersome-mediated delivery of doxorubicin into tumor sites enhances the therapeutic efficacy and reduces cardiotoxic effects. J. Control. Release.

[8] Discher D.E., Eisenberg A. (2002). Polymer Vesicles. Science.

[9] Le Meins J.F., Sandre O., Lecommandoux S. (2011). Recent trends in the tuning of polymersomes’ membrane properties. Eur. Phys. J. E.

[10] Marguet M., Edembe L., Lecommandoux S. (2012). Polymersomes in Polymersomes: Multiple Loading and Permeability Control. Angew. Chem..

[11] Zheng C., Qiu L., Zhu K. (2009). Novel polymersomes based on amphiphilic graft polyphosphazenes and their encapsulation of water-soluble anti-cancer drug. Polymer.

[12] Lee J.S., Feijen J. (2012). Polymersomes for drug delivery: Design, formation and characterization. J. Control. Release.

[13] Messager L., Gaitzsch J., Chierico L., Battaglia G. (2014). Novel aspects of encapsulation and delivery using polymersomes. Curr. Opin. Pharmacol..

[14] Martin C., Dolmazon E., Moylan K., Fowley C., McHale A.P., Callan J.F., Callan B. (2015). A charge neutral, size tuneable polymersome capable of high biological encapsulation efficiency and cell permeation. Int. J. Pharm..

[15] Levine D.H., Ghoroghchian P.P., Freudenberg J., Zhang G., Therien M.J., Greene M.I., Hammer D.A., Murali R. (2008). Polymersomes: A new multi-functional tool for cancer diagnosis and therapy. Methods.

[16] Walvekar P., Gannimani R., Salih M., Makhathini S., Mocktar C., Govender T. (2019). Self-assembled oleylamine grafted hyaluronic acid polymersomes for delivery of vancomycin against methicillin resistant Staphylococcus aureus (MRSA). Colloids Surfaces B Biointerfaces.

[17] Lefley J., Waldron C., Becer C.R. (2020). Macromolecular design and preparation of polymersomes. Polym. Chem..

[18] Rideau E., Dimova R., Schwille P., Wurm F.R., Landfester K. (2018). Liposomes and polymersomes: A comparative review towards cell mimicking. Chem. Soc. Rev..

[19] Foster J.C., Varlas S., Couturaud B., Jones J.R., Keogh R., Mathers R.T., O’Reilly R.K. (2018). Predicting Monomers for Use in Polymerization-Induced Self-Assembly. Angew. Chem. Int. Ed..

[20] Couturaud B., Georgiou P., Varlas S., Jones J., Arno M.C., Foster J., O’Reilly R.K. (2019). Poly(Pentafluorophenyl Methacrylate)-Based Nano-Objects Developed by Photo-PISA as Scaffolds for Post-Polymerization Functionalization. Macromol. Rapid Commun..

[21] Khor S.Y., Quinn J.F., Whittaker M.R., Truong N.P., Davis T.P. (2019). Controlling Nanomaterial Size and Shape for Biomedical Applications via Polymerization-Induced Self-Assembly. Macromol. Rapid Commun..

[22] Phan H., Cossutta M., Houppe C., Le Cœur C., Prevost S., Cascone I., Courty J., Penelle J., Couturaud B. (2022). Polymerization-Induced Self-Assembly (PISA) for in situ drug encapsulation or drug conjugation in cancer application. J. Colloid Interface Sci..

[23] Tan J., Liu D., Zhang X., Huang C., He J., Xu Q., Li X., Zhang L. (2017). Facile preparation of hybrid vesicles loaded with silica nanoparticles via aqueous photoinitiated polymerization-induced self-assembly. RSC Adv..

[24] Varlas S., Foster J.C., Georgiou P.G., Keogh R., Husband J.T., Williams D.S., O’Reilly R.K. (2019). Tuning the membrane permeability of polymersome nanoreactors developed by aqueous emulsion polymerization-induced self-assembly. Nanoscale.

[25] Phan H., Taresco V., Penelle J., Couturaud B. (2021). Polymerisation-induced self-assembly (PISA) as a straightforward formulation strategy for stimuli-responsive drug delivery systems and biomaterials: Recent advances. Biomater. Sci..

[26] Meng F., Zhong Z., Feijen J. (2009). Stimuli-Responsive Polymersomes for Programmed Drug Delivery. Biomacromolecules.

[27] Hu X., Zhang Y., Xie Z., Jing X., Bellotti A., Gu Z. (2017). Stimuli-Responsive Polymersomes for Biomedical Applications. Biomacromolecules.

[28] Chakraborty S., Ray D., Aswal V.K., Ghosh S. (2018). Multi-Stimuli-Responsive Directional Assembly of an Amphiphilic Donor-Acceptor Alternating Supramolecular Copolymer. Chem. A Eur. J..

[29] Ferrero C., Casas M., Caraballo I. (2022). Redox-Responsive Polymersomes as Smart Doxorubicin Delivery Systems. Pharmaceutics.

[30] Waris G., Ahsan H. (2006). Reactive oxygen species: Role in the development of cancer and various chronic conditions. J. Carcinog..

[31] Liou G.-Y., Storz P. (2010). Reactive oxygen species in cancer. Free Radic. Res..

[32] Kembro J.M., Cortassa S., Aon M.A. (2012). Mitochondrial Reactive Oxygen Species (ROS) and Arrhythmias. Systems Biology of Free Radicals and Antioxidants.

[33] Trachootham D., Alexandre J., Huang P. (2009). Targeting cancer cells by ROS-mediated mechanisms: A radical therapeutic approach?. Nat. Rev. Drug Discov..

[34] Ke W., Li J., Mohammed F., Wang Y., Tou K., Liu X., Wen P., Kinoh H., Anraku Y., Chen H. (2019). Therapeutic Polymersome Nanoreactors with Tumor-Specific Activable Cascade Reactions for Cooperative Cancer Therapy. ACS Nano.

[35] Hu X., Yu J., Qian C., Lu Y., Kahkoska A.R., Xie Z., Jing X., Buse J.B., Gu Z. (2017). H2O2-Responsive Vesicles Integrated with Transcutaneous Patches for Glucose-Mediated Insulin Delivery. ACS Nano.

[36] Zhou Q., Wang Y., Li X., Lu N., Ge Z. (2021). Polymersome Nanoreactor-Mediated Combination Chemodynamic-Immunotherapy via ROS Production and Enhanced STING Activation. Adv. Ther..

[37] Phan H., Cavanagh R., Destouches D., Vacherot F., Brissault B., Taresco V., Penelle J., Couturaud B. (2022). H_2_O_2_-Responsive Nanocarriers Prepared by RAFT-Mediated Polymerization-Induced Self-Assembly of *N*-(2-(Methylthio)ethyl)acrylamide for Biomedical Applications. ACS Appl. Polym. Mater..

[38] Julyan P.J., Seymour L.W., Ferry D.R., Daryani S., Boivin C.M., Doran J., David M., Anderson D., Christodoulou C., Young A.M. (1999). Preliminary clinical study of the distribution of HPMA copolymers bearing doxorubicin and galactosamine. J. Control. Release.

[39] Ulbrich K., Šubr V. (2010). Structural and chemical aspects of HPMA copolymers as drug carriers. Adv. Drug Deliv. Rev..

[40] Anane-Adjei A.B., Fletcher N.L., Cavanagh R.J., Houston Z.H., Crawford T., Pearce A.K., Taresco V., Ritchie A.A., Clarke P., Grabowska A.M. (2022). Synthesis, characterisation and evaluation of hyperbranched *N*-(2-hydroxypropyl) methacrylamides for transport and delivery in pancreatic cell lines in vitro and in vivo. Biomater. Sci..

[41] Scales C.W., Vasilieva Y.A., Convertine A.J., Lowe A.B., McCormick C.L. (2005). Direct, Controlled Synthesis of the Nonimmunogenic, Hydrophilic Polymer, Poly(*N*-(2-hydroxypropyl)methacrylamide) via RAFT in Aqueous Media. Biomacromolecules.

[42] Zwicke G.L., Mansoori G.A., Jeffery C.J. (2012). Utilizing the folate receptor for active targeting of cancer nanotherapeutics. Nano Rev..

[43] Fernández M., Javaid F., Chudasama V. (2018). Advances in targeting the folate receptor in the treatment/imaging of cancers. Chem. Sci..

[44] Lu Y., Sega E., Leamon C.P., Low P.S. (2004). Folate receptor-targeted immunotherapy of cancer: Mechanism and therapeutic potential. Adv. Drug Deliv. Rev..

[45] Sosnik A. (2018). From the “Magic Bullet” to Advanced Nanomaterials for Active Targeting in Diagnostics and Therapeutics.

[46] Bellotti E., Cascone M.G., Barbani N., Rossin D., Rastaldo R., Giachino C., Cristallini C. (2021). Targeting Cancer Cells Overexpressing Folate Receptors with New Terpolymer-Based Nanocapsules: Toward a Novel Targeted DNA Delivery System for Cancer Therapy. Biomedicines.

[47] Jawahar N., De A., Jubee S., Reddy E.S. (2020). Folic acid-conjugated raloxifene hydrochloride carbon nanotube for targeting breast cancer cells. Drug Dev. Res..

[48] Bauri K., Narayanan A., Haldar U., De P. (2015). Polymerization-induced self-assembly driving chiral nanostructured materials. Polym. Chem..

[49] Lowe A.B. (2016). RAFT alcoholic dispersion polymerization with polymerization-induced self-assembly. Polymer.

[50] Blanazs A., Ryan A.J., Armes S.P. (2012). Predictive Phase Diagrams for RAFT Aqueous Dispersion Polymerization: Effect of Block Copolymer Composition, Molecular Weight, and Copolymer Concentration. Macromolecules.

[51] Blanazs A., Madsen J., Battaglia G., Ryan A.J., Armes S.P. (2011). Mechanistic Insights for Block Copolymer Morphologies: How Do Worms Form Vesicles?. J. Am. Chem. Soc..

[52] Figg C.A., Carmean R.N., Bentz K.C., Mukherjee S., Savin D.A., Sumerlin B.S. (2017). Tuning Hydrophobicity to Program Block Copolymer Assemblies from the Inside Out. Macromolecules.

[53] Baddam V., Välinen L., Tenhu H. (2021). Thermoresponsive Polycation-Stabilized Nanoparticles through PISA. Control of Particle Morphology with a Salt. Macromolecules.

[54] Cunningham V.J., Alswieleh A.M., Thompson K.L., Williams M., Leggett G.J., Armes S.P., Musa O.M. (2014). Poly(glycerol monomethacrylate)–Poly(benzyl methacrylate) Diblock Copolymer Nanoparticles via RAFT Emulsion Polymerization: Synthesis, Characterization, and Interfacial Activity. Macromolecules.

[55] de la Haye J.L., Zhang X., Chaduc I., Brunel F., Lansalot M., D’Agosto F. (2016). The Effect of Hydrophile Topology in RAFT-Mediated Polymerization-Induced Self-Assembly. Angew. Chem. Int. Ed..

[56] Blackman L.D., Varlas S., Arno M.C., Fayter A., Gibson M.I., O’reilly R.K. (2017). Permeable Protein-Loaded Polymersome Cascade Nanoreactors by Polymerization-Induced Self-Assembly. ACS Macro Lett..

[57] Miller A.J., Pearce A.K., Foster J.C., O’Reilly R.K. (2021). Probing and Tuning the Permeability of Polymersomes. ACS Central Sci..

[58] Xu S., Ng G., Xu J., Kuchel R.P., Yeow J., Boyer C. (2017). 2-(Methylthio)ethyl Methacrylate: A Versatile Monomer for Stimuli Responsiveness and Polymerization-Induced Self-Assembly in the Presence of Air. ACS Macro Lett..

[59] Deng Y., Chen H., Tao X., Cao F., Trépout S., Ling J., Li M.-H. (2019). Oxidation-Sensitive Polymersomes Based on Amphiphilic Diblock Copolypeptoids. Biomacromolecules.

[60] Sobotta F.H., Kuchenbrod M.T., Gruschwitz F.V., Festag G., Bellstedt P., Hoeppener S., Brendel J.C. (2021). Tuneable Time Delay in the Burst Release from Oxidation-Sensitive Polymersomes Made by PISA. Angew. Chem. Int. Ed..

[61] Xu L., Zhao M., Gao W., Yang Y., Zhang J., Pu Y., He B. (2019). Polymeric nanoparticles responsive to intracellular ROS for anticancer drug delivery. Colloids Surfaces B Biointerfaces.

[62] Yoo J., Rejinold N.S., Lee D.Y., Jon S., Kim Y.-C. (2017). Protease-activatable cell-penetrating peptide possessing ROS-triggered phase transition for enhanced cancer therapy. J. Control. Release.

[63] de Gracia Lux C., Joshi-Barr S., Nguyen T., Mahmoud E., Schopf E., Fomina N., Almutairi A. (2012). Biocompatible Polymeric Nanoparticles Degrade and Release Cargo in Response to Biologically Relevant Levels of Hydrogen Peroxide. J. Am. Chem. Soc..

[64] Truong N.P., Dussert M.V., Whittaker M.R., Quinn J.F., Davis T.P. (2015). Rapid synthesis of ultrahigh molecular weight and low polydispersity polystyrene diblock copolymers by RAFT-mediated emulsion polymerization. Polym. Chem..

[65] Larnaudie S.C., Brendel J.C., Romero-Canelón I., Sanchez-Cano C., Catrouillet S., Sanchis J., Coverdale J.P.C., Song J.-I., Habtemariam A., Sadler P.J. (2018). Cyclic Peptide–Polymer Nanotubes as Efficient and Highly Potent Drug Delivery Systems for Organometallic Anticancer Complexes. Biomacromolecules.

[66] Ulbrich K., Šubr V., Strohalm J., Plocová D., Jelínková M., Říhová B. (2000). Polymeric drugs based on conjugates of synthetic and natural macromolecules: I. Synthesis and physico-chemical characterisation. J. Control. Release.

[67] Chytil P., Etrych T., Kříž J., Šubr V., Ulbrich K. (2010). N-(2-Hydroxypropyl)methacrylamide-based polymer conjugates with pH-controlled activation of doxorubicin for cell-specific or passive tumour targeting. Synthesis by RAFT polymerisation and physicochemical characterisation. Eur. J. Pharm. Sci..

[68] Wang X., Ma Q., Wen C., Gong T., Li J., Liang W., Li M., Wang Y., Guo R. (2021). Folic acid and deoxycholic acid derivative modified Fe_3_O_4_ nanoparticles for efficient pH-dependent drug release and multi-targeting against liver cancer cells. RSC Adv..

[69] Lee S.J., Shim Y.-H., Oh J.-S., Jeong Y.-I., Park I.-K., Lee H.C. (2015). Folic-acid-conjugated pullulan/poly(DL-lactide-co-glycolide) graft copolymer nanoparticles for folate-receptor-mediated drug delivery. Nanoscale Res. Lett..

