# Supplementary material for: Synthesis of Multifunctional Polymersomes Prepared by Polymerization-Induced Self-Assembly
Source: Polymers (Basel). 2023 Jul 17;15(14):3070. doi: 10.3390/polym15143070 (PMC10383388; doi:10.3390/polym15143070)
Supplement: Supplementary file 1 [file polymers-15-03070-s001.zip › polymers-2477946-supplementary.pdf]

polymers

MDPI

Supplementary Materials

Synthesis of Multifunctional Polymersomes Prepared by Polymerization-Induced Self-Assembly

Hien Phan [1,2], Robert Cavanagh [3], Philippa Jacob [2], Damien Destouches [4], Francis Vacherot [4], Benedetta Brugnoli [2,5], Steve Howdle [2], Vincenzo Taresco [2] and Benoit Couturaud [1,*]

[1] University Paris Est Créteil, CNRS, Institut de Chimie et des Matériaux Paris-Est (ICMPE), UMR 7182, 2 Rue Henri Dunant, 94320 Thiais, France; thi-dieu-hien.phan@cnrs.fr (H.P.);
[2] School of Chemistry, University of Nottingham, University Park, Nottingham NG7 2RD, UK; philippa.jacob@nottingham.ac.uk (P.J.); benedetta.brugnoli@uniroma1.it (B.B.); steve.howdle@nottingham.ac.uk (S.H.); vincenzo.taresco@nottingham.ac.uk (V.T.)
[3] School of Medicine, University of Nottingham, University Park, Nottingham NG7 2RD, UK; robert.cavanagh@nottingham.ac.uk
[4] University Paris Est Créteil, TRePCa, 94010 Créteil, France; damien.destouches@u-pec.fr (D.D.); francis.vacherot@u-pec.fr (F.V.)
[5] Department of Chemistry, Sapienza University of Rome, Piazzale Aldo Moro 5, 00185 Rome, Italy
* Correspondence: benoit.couturaud@cnrs.fr

Citation: Phan, H.; Cavanagh, R.; Jacob, P.; Destouches, D.; Vacherot, F.; Brugnoli, B.; Howdle, S.; Taresco, V.; Couturau, B. Synthesis of Multifunctional Polymersomes Prepared by Polymerization-Induced Self-Assembly. *Polymers* **2023**, *15*, x. https://doi.org/10.3390/xxxxx

Academic Editors: Arn Mignon, Andrea Sorrentino

Received: 14 June 2023
Revised: 11 July 2023
Accepted: 12 July 2023
Published: date

Copyright: © 2023 by the authors. Licensee MDPI, Basel, Switzerland. This article is an open access article distributed under the terms and conditions of the Creative Commons Attribution (CC BY) license (https://creativecommons.org/licenses/by/4.0/).

1. Instrumentation

1.1. Polymer characterization

Molecular weight determination of the precursor copolymers was obtained using size exclusion chromatography (SEC) in DMF as the eluent. SEC analyses were performed on a Shimadzu instrument fitted with mixed-C columns and RI. Molecular weight distributions (M_w/M_n) were calculated using poly(methyl methacrylate) (PMMA) standards. All ^{1}H, ^{13}C NMR spectra were recorded on a Bruker 400 MHz FT NMR spectrometer. Samples were prepared in deuterated dimethyl sulfoxide-d$_6$ (DMSO-d6) and their signals referenced to residual non-deuterated signals of the solvent. The spectra were analyzed using MestReNova 12.0.4 (Mestrelab Research S.L.). Absorbance readings were performed by using UV-Vis Spectrophotometer of the Agilent Cary 60.

1.2. Particle characterization

Transmission electron microscopy (TEM)

Dry-state transmission electron microscopy (TEM) imaging was performed on either FEI TECNAI F20 microscope at an S3 acceleration voltage of 200 kV. All aqueous samples were diluted with deionized water and then deposited onto formvar-coated copper grids. Samples were directly diluted with milliQ water at the ratio of 1:100 and then deposited onto graphene oxide-coated copper grids. After roughly 1 min, excess sample was blotted from the grid and the grid was stained with an aqueous 1 wt% uranyl acetate (UA) solution for 1 min prior to blotting, drying and microscopic analysis. For determination of the average particles' size at least 100 particles were analyzed in each case.

Dynamic Light Scattering (DLS)

The intensity-average sphere equivalent diameter of diblock copolymer nano-objects was determined at 25 °C by DLS using a Malvern Zetasizer Nano S instrument via the Stokes–Einstein equation, which assumes perfectly monodisperse, non-interacting spheres. All measurements were prepared at 1 mg/mL in water. Z-average hydrodynamic diameters (D_Z) were calculated from 3 repeat measurements.

2. Supporting results

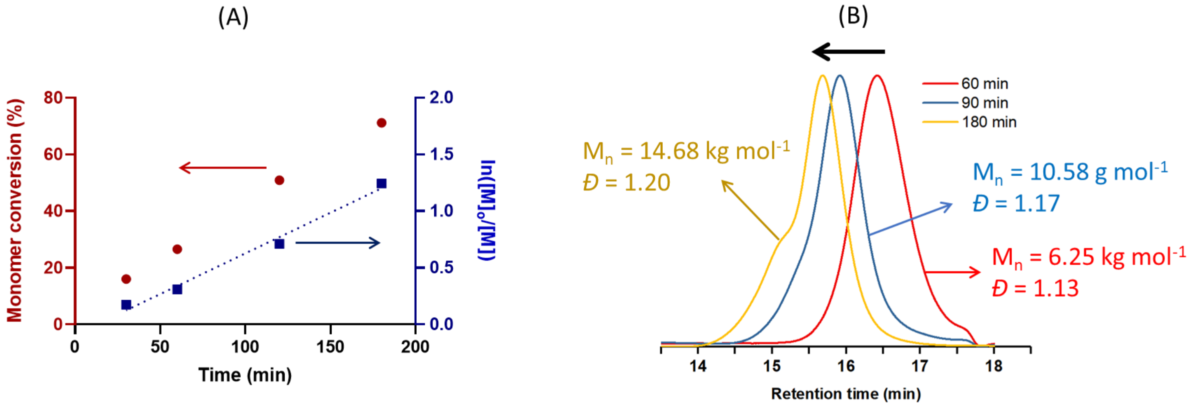

Figure S1. Kinetics of the RAFT polymerization of HPMAm in acetate buffer (pH 5.5)/dioxane (4/1), yielding $P(HPMAm)_{43}$ homopolymer at 180 min. (A) Monomer conversion % (red) and first-order kinetic plots (blue) as a function of time; (B) SEC chromatogram at 60, 90 and 180 min using DMF and PMMA standard.

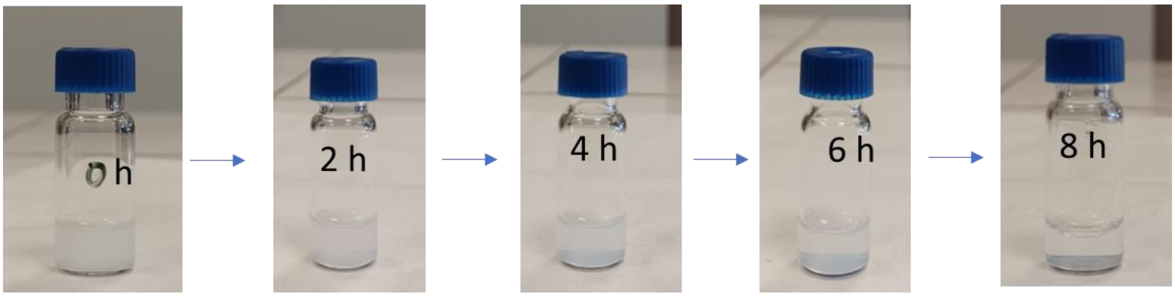

Figure S2. Color change from turbidity gradually to transparency of $P(HPMAm)_{43}$-b-$P(MTEAM)_{300}$ polymersomes (2 mg mL^{-1}) when incubated in 10 mM H_2O_2 over time.

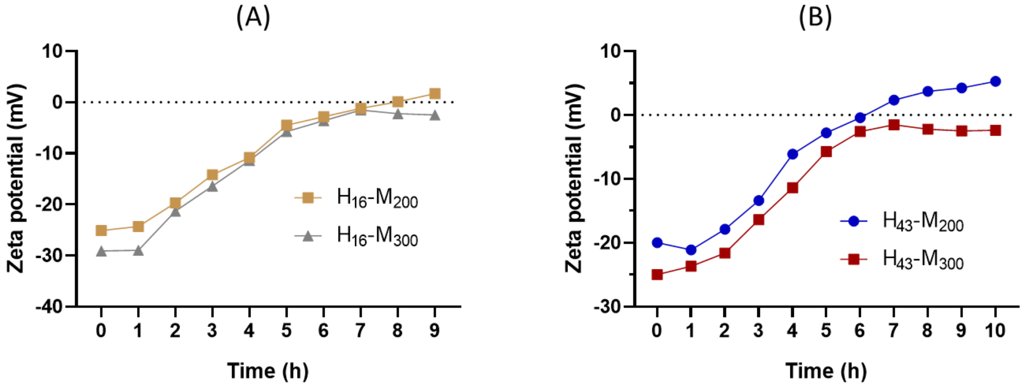

Figure S3. Zeta potentials (ZPs) of (A) $P(HPMAm)_{16}$-b-$P(MTEAM)_x$ (H_{16}-M_x) and (B) $P(HPMAm)_{43}$-b-$P(MTEAM)_x$ (H_{43}-M_x) (x = 200, 300) polymersomes at 1 mg mL^{-1} during the treatment with 10 mM H_2O_2.
